# Estimating 10-year risk of lung and breast cancer by occupation in Switzerland

**DOI:** 10.3389/fpubh.2023.1137820

**Published:** 2023-03-23

**Authors:** Bernadette Wilhelmina Antonia van der Linden, Nicolas Bovio, Patrick Arveux, Yvan Bergeron, Jean-Luc Bulliard, Evelyne Fournier, Simon Germann, Isabelle Konzelmann, Manuela Maspoli, Elisabetta Rapiti, Arnaud Chiolero, Irina Guseva Canu

**Affiliations:** ^1^Population Health Laboratory (#PopHealthLab), University of Fribourg, Fribourg, Switzerland; ^2^Fribourg Cancer Registry, Fribourg, Switzerland; ^3^Center for Primary Care and Public Health (Unisanté), University of Lausanne, Lausanne, Switzerland; ^4^Neuchâtel and Jura Cancer Registry, Neuchâtel, Switzerland; ^5^Geneva Cancer Registry, University of Geneva, Geneva, Switzerland; ^6^Valais Cancer Registry, Valais Health Observatory, Sion, Switzerland; ^7^Institute of Primary Health Care (BIHAM), University of Bern, Bern, Switzerland; ^8^School of Population and Global Health, McGill University, Montréal, Canada

**Keywords:** breast cancer, lung cancer, occupation, risk communication, Switzerland

## Abstract

**Introduction:**

Lung and breast cancer are important in the working-age population both in terms of incidence and costs. The study aims were to estimate the 10-year risk of lung and breast cancer by occupation and smoking status and to create easy to use age-, and sex-specific 10-year risk charts.

**Methods:**

New lung and breast cancer cases between 2010 and 2014 from all 5 cancer registries of Western Switzerland, matched with the Swiss National Cohort were used. The 10-year risks of lung and breast cancer by occupational category were estimated. For lung cancer, estimates were additionally stratified by smoking status using data on smoking prevalence from the 2007 Swiss Health Survey.

**Results:**

The risks of lung and breast cancer increased with age and were the highest for current smokers. Men in elementary professions had a higher 10-year risk of developing lung cancer compared to men in intermediate and managerial professions. Women in intermediate professions had a higher 10-year risk of developing lung cancer compared to elementary and managerial professions. However, women in managerial professions had the highest risk of developing breast cancer.

**Discussion:**

The 10-year risk of lung and breast cancer differs substantially between occupational categories. Smoking creates greater changes in 10-year risk than occupation for both sexes. The 10-year risk is interesting for both patients and professionals to inform choices related to cancer risk, such as screening and health behaviors. The risk charts can also be used as public health indicators and to inform policies to protect workers.

## 1. Introduction

Lung cancer, in men and women, and breast cancer, in women, are important in the working-age population both in terms of incidence and costs (1). In Switzerland, between 2013 and 2017, lung cancer was the second most common cancer diagnosed in men and the third in women and the leading cause of cancer deaths in men and the second in women (2). In the same period, breast cancer was the most common type of cancer diagnosed in women, but had a lower mortality rate than lung cancer (2). Both lung and breast cancer were more often diagnosed in French-speaking (Western) Switzerland than German-speaking Switzerland (2).

Associations of lifestyle-related factors with cancer risk have been explored in the literature, but accurate estimates of the cancer burden associated with occupations is lacking. Assessing the effect of occupation on cancer risk is challenging due to data availability and quality, particularly on measurements and levels of exposures, and information on employment. Burden estimates are only available for a few countries and are not directly comparable (3–5). Occupation can be considered as proxy of occupational exposures and working conditions that may result in the development of occupational cancers (3, 6). Occupational cancers are those that are wholly or partly caused by exposure to carcinogens or circumstances at work (7). In particular, several carcinogens that can potentially cause lung cancer have been identified in occupational settings, such as arsenic, asbestos, cadmium, and diesel fumes (1, 8–11) while ionizing radiation, some chemicals, and night work are suggested to be related to female breast cancer (12–15). An estimated 10 and 5% of lung cancer deaths among men and women, respectively, could be attributable to exposure to occupational carcinogens (11).

Accounting for smoking patterns is key to understanding the burden of occupational cancer. Smoking prevalence varies by occupation due to, for example, sociodemographic and employment-related factors (16). Previous studies found higher smoking rates in blue-collar occupations, such as construction and extraction, compared to managers (17, 18). Since smoking is a major cause of lung cancer, trends in lung cancer incidence follow trends in smoking behavior. In Switzerland, smoking prevalence among men peaked in the 1950's and among women in the 1970's, which was followed by a lung cancer incidence peak among men in the 1980's and an increase since the 1970's among women (19–21).

To our knowledge, there is scarce evidence on the association of occupation with cancers in Switzerland (8, 10). One study among Swiss men showed increased cancer risk for several occupations, with results varying by cancer site (22). However, this study had several limitations, most notably the quality of occupational definition. In addition, for health decision making, it is useful to estimate an individual's risk of disease in the future. The cumulative 10-year risk, which is the probability for a given individual of developing a given disease in the next 10 years, is an appropriate metric to do so (23, 24).

Our first aim was therefore to estimate the age-, and sex-specific cumulative 10-year risk of both lung cancer, in men and women, and breast cancer, in women, in Western Switzerland, by occupation, and, for lung cancer, also by smoking status. Our second aim was to create easy to interpret risk charts for lung and breast cancer using the previously calculated risk estimates.

## 2. Methods

### 2.1. Data sources

New lung and breast cancer cases registered by all five cancer registries in Western Switzerland (Fribourg, Geneva, Neuchatel-Jura, Valais, and Vaud) between 2010 and 2014 were used. Information on occupation and vital status were retrieved from the Swiss National Cohort (SNC) and were matched with the data from the cancer registries. The SNC covers an estimated 98.6% of the population and is based on data from the 1990 and 2000 federal censuses (25). Smoking prevalence, stratified by age and sex, was computed using data from the 2007 Swiss Health Survey (SHS). The SHS is a survey on the health status and determinants of health of the Swiss population aged 15 years and older.

Participants were eligible for the study if they were of working and transitional to retirement age (18 to 65 years at study entry) and were permanent residents of one of the Western Swiss cantons. In addition, they had to be included into the SNC either on December 4, 1990 (the date of the 1990 census) or on December 5, 2000 (the date of the 2000 census) and have information on occupation. End of follow-up was at the earliest date of: death, emigration, 85th birthday, or end of the study (December 31, 2014).

### 2.2. Outcomes

Lung cancer, in men and women, and breast cancer, in women, were the two primary outcomes. Only incident primary malignant cancers were considered and were identified based on the data from the cancer registries, coded using the International Classification of Diseases for Oncology (ICD-O), 3rd edition: C33–C34 for lung cancer and C50 for breast cancer. Using probabilistic linkage, the Institute of Social and Preventive Medicine of the University of Bern linked the registries' data with SNC data. Almost all lung and breast cancer could be linked with the SNC data (94.4%).

### 2.3. Occupation

Occupational status was categorized using the International Standard Classification of Occupations version 1988 (ISCO-88). This classification was used in the 1990 and 2000 censuses and coded using four-digit codes by the Swiss Federal Statistical Office. Based on this information, the following three categories were constructed: workers in (A) agriculture, industry, crafts, and elementary professions; (B) intermediate professions, administrative employees, personal service and personnel; and (C) managerial, intellectual or scientific professions. In this study, category A was labeled as “elementary professions,” category B as “intermediate professions,” and category C as “managerial professions” (26).

### 2.4. Smoking

Sex-, age-, and time-period specific prevalence of never, former, and current smoking in Switzerland were used to compute risks. To estimate the risk of lung cancer by smoking status, national smoking prevalence data stratified by age and sex were used. Smoking status was defined as a categorical variable: never smokers, former smokers, and current smokers, based on the questions “Do you currently smoke?” and “Have you ever smoked regularly for more than 6 months?” in the 2007 SHS. Using the 2007 survey provides the most recent prevalence estimates before cancer diagnosis.

### 2.5. Calculations

The 10-year risks of lung and breast cancer at age 35, 45, 55, 65, and 75 between the years 2010 to 2014, by occupation, were estimated following the studies by Bruder et al. and Woloshin et al. using the formulas below (23, 24). The 10-year risk of lung cancer at age A was computed by the difference between the cumulative risk until age A + 10 years and the cumulative risk until age A. For an individual aged 35 this would mean the risk of getting lung cancer between the ages of 35 and 44 was calculated as the cumulative risk of having lung cancer at age 44 minus the cumulative risk at age 35. Separate charts were created for men and women to take the influence of sex into account. As lung cancer is highly related to smoking habits, the risk charts for lung cancer were stratified by smoking status: current, former, never smoker.

#### 1. Calculation of age-, sex-, occupation-specific incidence rate

Age-, sex-, cause-specific lung cancer risk = all men of a specific age and occupation who were diagnosed with lung cancer between 2010 and 2014 divided by the total number of men of that age in the population and occupation between 2010 and 2014.

#### 2. Calculation of age-, sex-, occupation-specific incidence rate among never smokers


$$\begin{array}{l} {\text{Risk}}_{\text{never smokers}}{\text{= risk}}_{\text{whole population}}\text{/}({\text{prevalence}}_{\text{never}\;\text{smokers}}\\ {\text{ + prevalence}}_{\text{current smokers}}\text{x }{\text{RR}}_{\text{current}\;\text{smokers}}\;\\ {\text{ + prevalence}}_{\text{former smokers}}\text{x }{\text{RR}}_{\text{former}\;\text{smokers}}) \end{array}$$

The Swiss Health Survey was used to compute the prevalence of each smoking strata by age, sex, and time. The relative risk (RR) of lung cancer associated with current and former smoking compared to never smoking was used: RR = 6.57 for current smokers below 60 years of age, 9.62 for current smokers between 60 and 69, 9.07 for current smokers 70 years or over, and 4.30 for former smokers of all ages (27).

#### 3. Calculation of age-, sex-, occupation-specific incidence rate among current smokers

For any given age group and sex:


$$\begin{array}{l} \text{Ris}{\text{k}}_{\text{current smokers}}=\text{ris}{\text{k}}_{\text{never smokers}}\text{ x R}{\text{R}}_{\text{currentsmokers}} \end{array}$$

#### 4. Calculation of age-, sex-, occupation-specific incidence rate among former smokers

For any given age group and sex:


$$\begin{array}{l} \text{Ris}{\text{k}}_{\text{former smokers}}=\text{ris}{\text{k}}_{\text{never smokers}}\text{ x R}{\text{R}}_{\text{formersmokers}} \end{array}$$

#### 5. Accumulation of the age-, sex-, occupation-specific incidence rates

To estimate 10-year risk for the age group starting at age X, accumulate the risks for age X until X+9.

### 2.6. Risk charts

**Figure 1:**
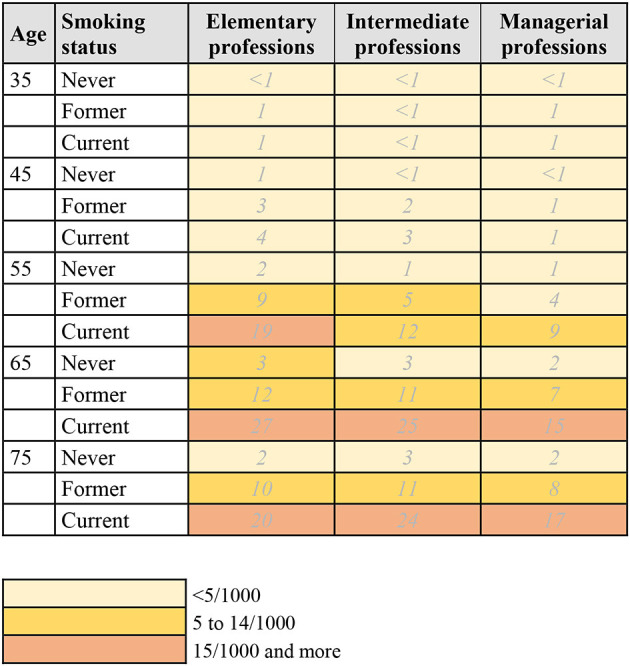
10-year risk chart of lung cancer for men by occupational group and smoking status. The chart indicates the number of men per 1,000 who will get lung cancer during the next 10 years, beginning at the indicated age. Elementary professions: agriculture, industry, crafts, elementary professions. Intermediate professions: intermediate professions, administrative employees, personal service and personnel. Managerial professions: managerial, intellectual, or scientific professions.

**Figure 2:**
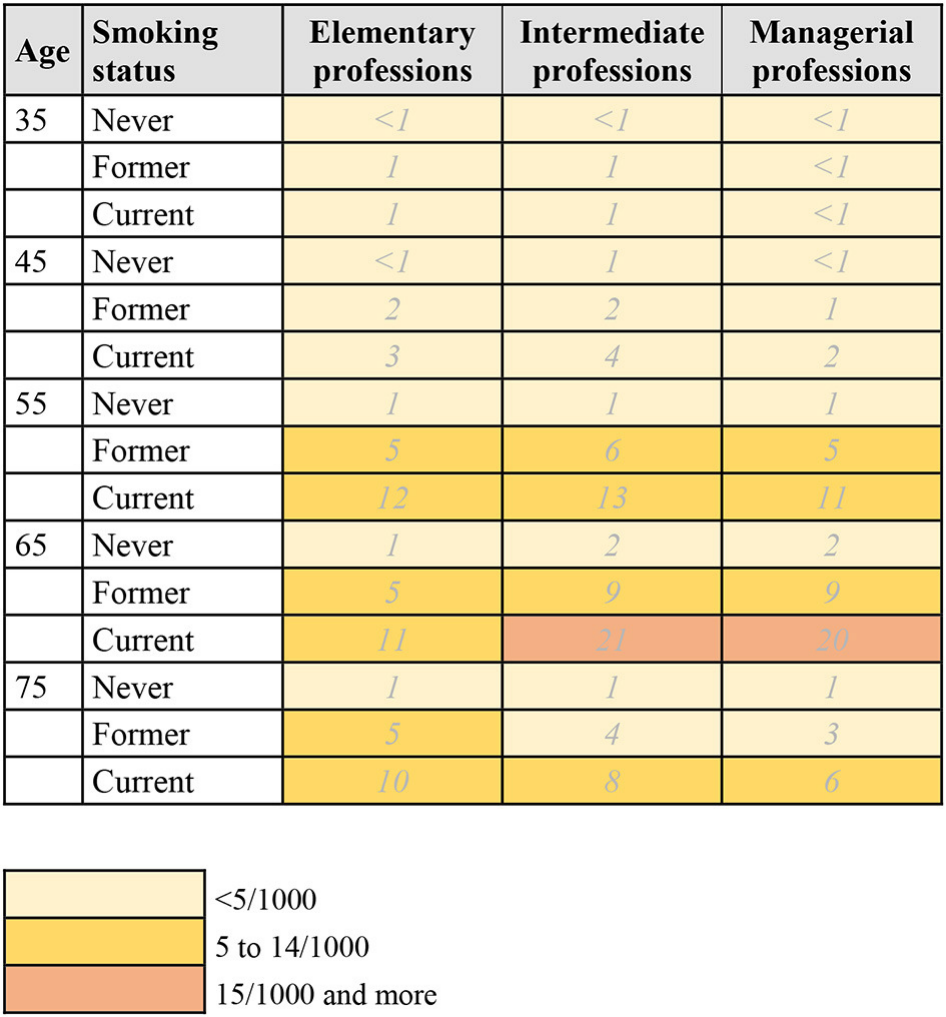
10-year risk chart of lung cancer for women by occupational group and smoking status. The chart indicates the number of women per 1,000 who will get lung cancer during the next 10 years, beginning at the indicated age. Elementary professions: agriculture, industry, crafts, elementary professions. Intermediate professions: intermediate professions, administrative employees, personal service and personnel. Managerial professions: managerial, intellectual, or scientific professions.

**Figure 3:**
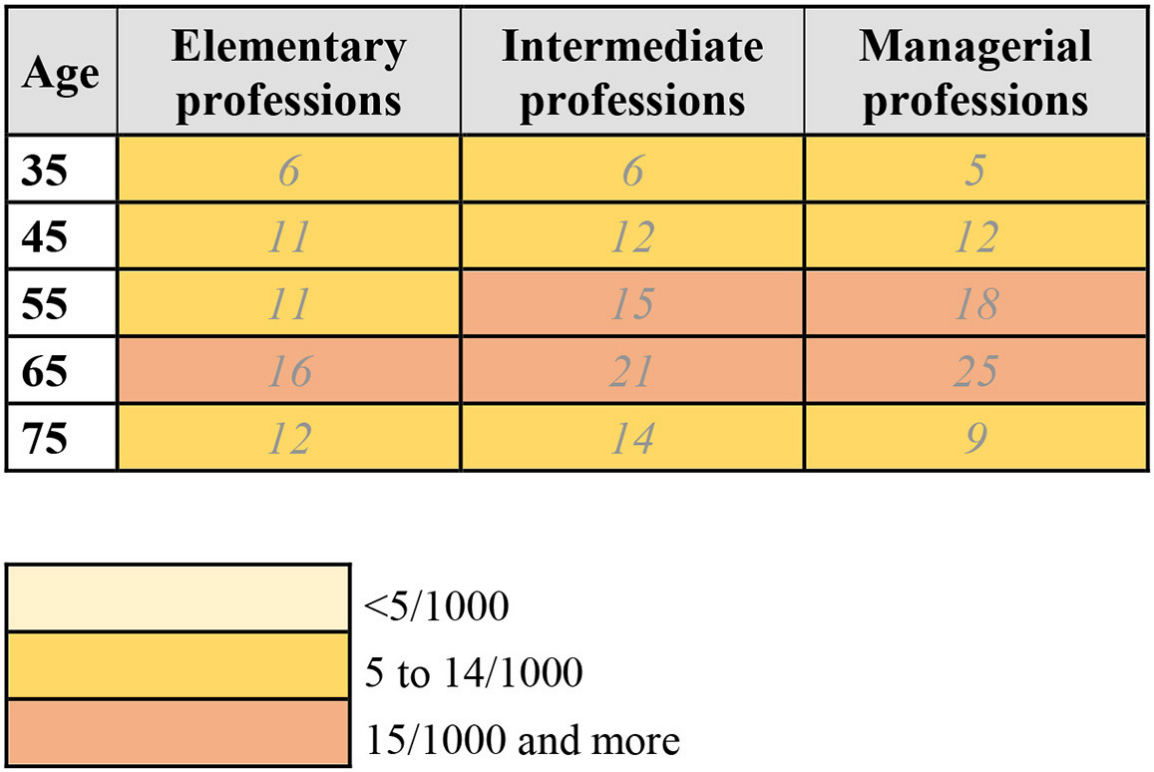
10-year risk chart of breast cancer for women by occupational group. The chart indicates the number of women per 1,000 who will get breast cancer during the next 10 years, beginning at the indicated age. Elementary professions: agriculture, industry, crafts, elementary professions. Intermediate professions: intermediate professions, administrative employees, personal service and personnel. Managerial professions: managerial, intellectual, or scientific professions.

Using the calculated 10-year risk estimates, risk charts for lung and breast cancer, by age group, occupational status, and smoking status were created in the following way: the charts (see Figures 1–3) show the number of individuals per 1,000 who will develop lung or breast cancer over the next 10 years, beginning at the indicated age. For each age category, from left to right, the risk for individuals in the elementary professions, intermediate professions, and in managerial professions are shown. The risk charts for lung cancer additionally show the risk by smoking status. Within each age category, the top row shows the risk for never smokers, the middle row for former smokers, and the bottom row for current smokers.

## 3. Results

In total, 3,090 men and 2,102 women were diagnosed with lung cancer and 7,045 women were diagnosed with breast cancer in the period 2010–2014 and were included in the study (Table 1). Most men were working in elementary professions and most women in intermediate professions. The prevalence of never smokers was the highest in the total population among both men and women, 43 and 58%, respectively.

**Table 1:** Participant characteristics 2010–2014.

	**Men lung cancer^a^**	**Women lung cancer^a^**	**Women breast cancer^a^**
Incidence rate/1,000	4	2	8
N elementary professions	548	59	261
N intermediate professions	370	406	1,869
N managerial professions	257	82	519
N other/missing	1,915	1,555	4,396
Prevalence of current smokers^b^	32.3%	23.6%	23.6%
Prevalence of former smokers^b^	24.3%	18.2%	18.2%
Prevalence of never smokers^b^	43.4%	58.2%	58.2%

a Total N (N other/missing profession) men lung cancer 3,090 (1,915), women lung cancer 2,102 (1,555), women breast cancer 7,045 (4,396).

b Total prevalence in the Swiss population for all ages, by sex.

### 3.1. Risk chart for lung cancer in men

Figure 1 and Supplementary material 1 show the risk chart of developing lung cancer for men. The overall pattern of risk within the next 10 years increases with every age group until age 75. In addition, men in elementary professions have the highest risk of developing lung cancer, at all ages. For example, in the age group 55–64, the risk estimate for men in elementary professions is 10/1,000, compared to 6/1,000 for men in intermediate professions and 5/1,000 for men in managerial jobs (Supplementary material 1). When also taking smoking status into account (Figure 1), current smokers have the highest risk of developing lung cancer. Looking at the age group 55–64 again, the risk estimates for men in elementary professions is 19/1,000 for current smokers, compared to 9/1,000 in former smokers and 2/1,000 in never smokers.

### 3.2. Risk chart for lung cancer in women

The risk chart for lung cancer in women is shown in Figure 2 and Supplementary material 2. As in men, the risk within the next 10 years increases with every age group until age 75. Women in intermediate professions have the highest risk of developing lung cancer between the ages of 45 and 75, before and after that, women in elementary professions have a higher risk. For example, in the age group 55–64, the risk estimate for women in intermediate professions is 5/1,000, compared to 4/1,000 for women in both elementary and managerial professions (Supplementary Material 2). This overall pattern persists when also taking smoking status into account (Figure 2), where current smokers have the highest risk of developing lung cancer. For women aged 55–64 in intermediate professions, the 10-year risk is 13/1,000 for current smokers, compared to 6/1,000 for former smokers and 1/1,000 for never smokers.

### 3.3. Risk chart for breast cancer in women

Figure 3 displays the risk chart for breast cancer in women. Like for lung cancer, the risk of developing breast cancer increases with every age group until age 75. Whereas, for lung cancer women in managerial professions had the lowest overall risk of developing lung cancer, this group has the highest risk of developing breast cancer between the ages of 45 and 65. The risk for women in elementary and intermediate professions is higher below the age of 45 and over the age of 75. For example, in the age group 65–74, the risk estimate for women in managerial professions is 25/1,000, compared to 21/1,000 for women in intermediate professions and 16/1,000 for women in elementary professions.

## 4. Discussion

Charts were created to estimate the sex-specific 10-year risk of both lung cancer, in men and women, and breast cancer, in women, in Western Switzerland, by occupation, and, for lung cancer, by smoking status. To the best of our knowledge, these are the first estimates of 10-year risk of developing lung or breast cancer taking occupational status into account. Results showed an overall increasing risk of lung and breast cancer by age group. Men in elementary and women in intermediate professions had the highest risk of developing lung cancer. Women in managerial professions had the highest risk of developing breast cancer. Smoking status creates greater change in 10-year risk than occupational status for both sexes. In addition, 10-year risk of lung cancer remains higher in men than women in Western Switzerland. In women, 10-year risk is higher for breast than lung cancer, except for current smokers.

The association between occupation and cancer can be direct through occupational exposures, but the occupational categories can also be viewed as a descriptor of socioeconomic status (28). Studies on socioeconomic status and cancer show similar results as our findings. Risk of lung cancer has been shown to be higher among lower socioeconomic status individuals (29–32). This socioeconomic gradient is likely due to differences in risk factors and exposures related to lung cancer, such as smoking, diet and occupational exposures (33–35). In women, a higher socioeconomic status is associated with a higher risk of breast cancer (36, 37). Possible explanations are diet, exercise and women's reproductive behaviors (38, 39).

A main strength of this study was the use of high-quality and exhaustive data from five cancer registries in Switzerland, with information on all cases of lung and breast cancer (40). This allowed for a reliable estimate of the incidence rates. We also computed up-to-date and easy to interpret risk graphs that are useful in risk communication and health decision making.

This study also has some limitations. First, more personalized risk estimates, taking other disease-specific and synergistic effects between risk factors into account, would be even more useful for reliable estimates. Non-occupational risk factors such as diet, physical activity, weight, socioeconomic status, and air pollution could be interesting to include in the calculations (11, 12). In our calculations we used estimates of the sur-risk due to smoking, which underestimates the risk of lung cancer. This will be in particular the case among workers in certain professions where exposure to certain lung carcinogens is more likely. However, this was not the aim of this study and would create complexity to the easy to interpret risk charts that already account for the most important risk factors. It could also be useful to estimate the 10-year risk of breast cancer in women by smoking status, as some studies found an association of passive and active smoking with higher risk of breast cancer (41). However, currently available risk estimates for exposure to tobacco and breast cancer differs by factors such as age at starting smoking relative to age at menarche and family history of breast cancer, and we did not have data available on this. In addition, accounting for smoking patterns is key when estimating lung cancer, but smoking is not the major cause of breast cancer. Another limitation is that the estimate of smoking status, was not directly available in the data. Smoking prevalence in Switzerland in 2007 by age group and sex was used as an estimate, which should be valid to some extent, but may have under- or overestimated the risk differences between never, former, and current smokers. Finally, only data from Western Switzerland was used to create the risk charts. Therefore, these charts may not give accurate estimates for the whole country. However, the aim of this paper was to estimate 10-year risk of lung and breast cancer by occupational status and to create easy to interpret risk charts.

The 10-year risk charts are useful for both patients and professionals to inform choices related to cancer risk, such as screening and health behaviors. The charts clearly show the magnitude related to certain risk factors, in this case smoking, which could facilitate health professionals in consultations about smoking cessation and informed health decision making (23). The risk charts can also be informative as public health indicators and for policies to protect workers. Adequate and clear risk communication about diseases such as cancer, that are often feared by individuals, is important. It can also be valuable to create these charts for other diseases to show an individual's risk of this disease, but also to put the risks of different diseases into context (23). Frequently used measures in public health, such as incidence and prevalence, are difficult for lay persons to understand and translate to their individual risk, whereas lifetime and 10-year risk seem to be more informative, intuitive, and easily understood (23, 24).

## 5. Conclusion

This is the first study that estimates the 10-year risk of developing lung or breast cancer by occupational status in Switzerland. Results indicate that men in elementary professions and women in intermediate professions have the highest risk of developing lung cancer. Women in the managerial professions have the highest risk of developing breast cancer.

Estimating an individual's probability of developing cancer in the next 10 years, i.e., the cumulative 10-year risk, is informative and easily understood by both individuals and health professionals (23, 24). However, these risk estimates need to be updated regularly to stay adequate, as health behaviors and incidence rates for lung and breast cancer change over time. We also recommend future larger studies to take synergistic effects between risk factors into account to have more precise estimates. When up to date and simple risk charts are available, they have the potential to aid in discussions about cancer risk as well as health decision making, such as screening or health behaviors, by highlighting the differences in 10-year risk between groups. In addition, by taking occupation into account, populations at high risk may be identified and awareness for the importance of occupational exposures and working conditions may be raised.

## Data availability statement

The datasets presented in this article are not readily available because due to the nature of this research, study participants could not agree for their data to be shared publicly. Supporting data is therefore not available in accordance with ethical and legal requirements. Requests to access the datasets should be directed to bernadette.vanderlinden@unifr.ch.

## Ethics statement

The studies involving human participants were reviewed and approved by Swissethics have approved the creation and use of this database for this project (N° de ref CER-VD 2018-02077). Written informed consent for participation was not required for this study in accordance with the national legislation and the institutional requirements.

## Author contributions

BL, NB, AC, and IG: conceptualization, validation, and methodology. NB: data curation. BL: formal analysis and writing—original draft preparation. NB, PA, YB, J-LB, EF, SG, IK, MM, ER, AC, and IG: writing—review and editing. All authors have read and agreed to the published version of the manuscript.
