# Supplementary material for: Estimating 10-year risk of lung and breast cancer by occupation in Switzerland
Source: Front Public Health. 2023 Mar 23;11:1137820. doi: 10.3389/fpubh.2023.1137820 (PMC10076749; doi:10.3389/fpubh.2023.1137820)
Supplement: Supplementary file 1 [file Data_Sheet_1.pdf]

Supplementary Material

Estimating 10-year risk of lung and breast cancer by occupation in Switzerland

Bernadette W.A. van der Linden[1,2*], Nicolas Bovio[3], Patrick Arveux[3], Yvan Bergeron[2], Jean-Luc Bulliard[3,4], Evelyne Fournier[5], Simon Germann[3], Isabelle Konzelmann[6], Manuela Maspoli[5], Elisabetta Rapiti[5], Arnaud Chiolero[1,6,7,8†], Irina Guseva Canu[3†]

***Correspondence:** Bernadette W.A. van der Linden: bernadette.vanderlinden@unifr.ch

Supplementary Material 1 10-year risk chart of lung cancer for men by occupational group

Age	Elementary professions	Intermediate professions	Managerial professions
**35**	*<1*	*<1*	*<1*
**45**	*3*	*2*	*1*
**55**	*10*	*6*	*5*
**65**	*12*	*11*	*7*
**75**	*8*	*10*	*7*

- <5/1000
- 5 to 14/1000
- 15/1000 and more

The chart indicates the number of men per 1000 who will get lung cancer during the next 10 years, beginning at the indicated age.

Elementary professions: agriculture, industry, crafts, elementary professions

Intermediate professions: intermediate professions, administrative employees, personal service and personnel

Managerial professions: managerial, intellectual, or scientific professions

Supplementary Material 2 10-year risk chart of lung cancer for women by occupational group

Age	Elementary professions	Intermediate professions	Managerial professions
**35**	*1*	*<1*	*<1*
**45**	*2*	*2*	*1*
**55**	*4*	*5*	*4*
**65**	*3*	*6*	*6*
**75**	*8*	*6*	*4*

	<5/1000
	5 to 14/1000
	15/1000 and more

The chart indicates the number of women per 1000 who will get lung cancer during the next 10 years, beginning at the indicated age.

Elementary professions: agriculture, industry, crafts, elementary professions

Intermediate professions: intermediate professions, administrative employees, personal service and personnel

Managerial professions: managerial, intellectual, or scientific professions
